# Supplementary figures and images for: COVID-19 reinfection in Liberia: Implication for improving disease surveillance
Source: PLoS One. 2022 Mar 24;17(3):e0265768. doi: 10.1371/journal.pone.0265768 (PMC8947140; doi:10.1371/journal.pone.0265768)

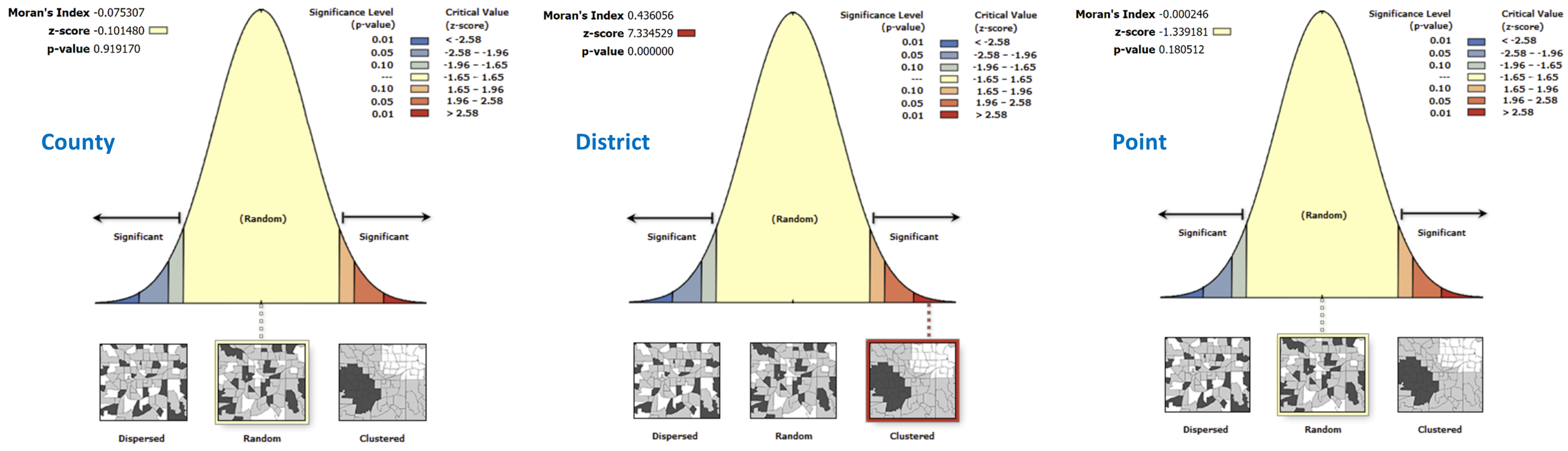

Supplement: S1 Fig — (TIF) [file pone.0265768.s003.tif]
