# Supplementary material for: COVID-19 reinfection in Liberia: Implication for improving disease surveillance
Source: PLoS One. 2022 Mar 24;17(3):e0265768. doi: 10.1371/journal.pone.0265768 (PMC8947140; doi:10.1371/journal.pone.0265768)
Supplement: Attachment — Submitted filename: Response to Reviewers.docx [file pone.0265768.s004.docx]

Liberia Field Epidemiology Training Program,

African Field Epidemiology Network (AFENET),

Monrovia, Liberia.

[gakpan@afenet.net](mailto:gakpan@afenet.net)

February 16, 2022

Siew Ann Cheong, Ph.D.,
Academic Editor,
PLOS ONE.

**RE:** COVID-19 reinfection in Liberia: implication for improving disease surveillance **[PONE-D-21-36066]**

Dear Dr. Cheong,

We are very grateful to you and the reviewers for the time and efforts you all put in to review our manuscript and for valuable suggestions to improve the understanding of the work.

We have revised the manuscript to address the points raised during the review process. Our point-by-point responses to all comments and suggestions are tabulated on the next page of this letter.

We hope you find our responses satisfactory and the manuscript now suitable for publication in PLOS ONE, even as we appreciate further suggestions where we failed to address observed concerns.

Sincerely,

**Godwin Akpan**

Geoinformation Systems and IT Specialist,

Liberia Field Epidemiology Training Program, AFENET.

[On behalf of all authors]

**Response to reviewer’s comments**

| **Section** | **Reviewers’ comments** | **Authors response** |
| --- | --- | --- |
| Editor’s comment |  |  |
|  | 1. Specifically, Reviewer 1 questioned how generalizable the results from a single county can be, and requested for an analysis of the spatial clustering of cases. Please address these, and other comments from the reviewers | Thank you for the thorough review of our manuscript and for useful suggestions to improve the understanding of the work. We have carefully gone through the comments and made modifications as suggested in the work. We have also responded to the comments here for ease of reference to the changes made in the work. We hope that the work is now acceptable for publication and will be available to respond to further clarification, if any. |
| Journal Requirements |  |  |
|  | 1. Please ensure that your manuscript meets PLOS ONE's style requirements, including those for file naming. The PLOS ONE style templates can be found at https://journals.plos.org/plosone/s/file?id=wjVg/PLOSOne_formatting_sample_main_body.pdf and https://journals.plos.org/plosone/s/file?id=ba62/PLOSOne_formatting_sample_title_authors_affiliations.pdf. | Thank you for the support link. We have used the attached link to format the manuscript to meet PLOS ONE’s style requirements. |
|  | 2. Please amend your current ethics statement to address the following concerns:  a) Did participants provide their written or verbal informed consent to participate in this study?  b) If consent was verbal, please explain  i) why written consent was not obtained  ii) how you documented participant consent, and  iii) whether the ethics committees/IRB approved this consent procedure. | Thank you for pointing out this. Written informed consent was obtained from the cases. This has been updated in the ethical consideration section of the work. ***[Lines 160 and 161]*** |
|  | 3. We note that Figures 2 and 4 in your submission contain map/satellite images which may be copyrighted. All PLOS content is published under the Creative Commons Attribution License (CC BY 4.0), which means that the manuscript, images, and Supporting Information files will be freely available online, and any third party is permitted to access, download, copy, distribute, and use these materials in any way, even commercially, with proper attribution. For these reasons, we cannot publish previously copyrighted maps or satellite images created using proprietary data, such as Google software (Google Maps, Street View, and Earth).  We require you to either (1) present written permission from the copyright holder to publish these figures specifically under the CC BY 4.0 license, or (2) remove the figures from your submission | Figures 2 and 4 were maps created by the authors for the work using ArcGIS Pro Version 2.8.2. They are not copyrighted materials, and so they do not need copyright permission. They can be published under the creative commons attribution license (CC BY 4.0) |
| Reviewer 1 |  |  |
|  | As mentioned, more than 80% cases occurred in one county and 86% cases of reinfection from the same county. The generalizability of this data for the whole country is questionable. | Thank you for these points. The authors did not set out to make generalizations but rather describe the patterns of reinfection observed in the routine surveillance data and make a case for providing laboratory capacity for genomic sequencing in the country. To address the concern raised, the authors have added a section to assess the spatial pattern of distribution of the cases ***[Lines 130-145]***.  It is also important to note that:  1. Montserrado County accounts for the majority of the population of Liberia's 5.2 million. Of the 3,476,608 population of Liberia based on the 2008 census, Montserrado County made up 1,118,241 (32.2%) of the 15 counties. https://www.lisgis.net/page_info.php?7d5f44532cbfc489b8db9e12e44eb820=MzQy  2. Considering the high rates (77%) of reinfection among [international travelers, the hub of travel is Montserrado with the only two international airports. Additionally, the dense population and high community transmission and traditional family and friends contacts in Montserrado means that those travelers also made contacts with others in Montserrado County  3. The authors have analyzed several data points and provided reports to key stakeholders that have shown that Montserrado County data can be generalizable to a greater extent to the rest of Liberia on several attributes, including testing, cases, contacts, hospitalization. |
|  | As data on geolocation was available, Moran’s Index should have been calculated - clustering for events? | An additional section to address this has been added, and it reads,  “To assess the overall distribution pattern of COVID-19 cases across the country – whether clustered, dispersed, or random, we used Spatial Autocorrelation (Global Moran's I). We used Cluster and Outlier Analysis (Anselin Local Moran's I) to identify the specific area and how the pattern exists. With the null hypothesis that the pattern is random or that no spatial autocorrelation is present in the transmission or distribution of the COVID-19 cases, we applied Global Moran's I in three different Input Feature Classes: county, district, and the location of individual cases (point). In the same vein, we applied Local Moran's I in two different Input Feature Classes: district and point.  To ascertain the influence of COVID-19 cases on nearby neighboring or distant cases (target feature), we selected an Inverse Distance Conceptualization of Spatial Relationships with Euclidean Distance method in the Global Moran's I tool. We also set the same parameters as in Global Moran's I for Local Moran's I, with Row standardization and 999 Permutations to improve the precision of the pseudo p-value and the random distribution of cases.” The results of the analysis have also been added to the work. ***[Lines 186-201]*** |
|  | Majority of reinfection cases were international travelers - How was international travel defined? | The authors defined international travelers as “citizens who visited and returned from other countries or non-citizens who came into the country either by land border crossing, sea or flight.” This has been added to the work. ***[Lines 80-82]*** |
|  | Details related to in country measures to mitigate spread of COVID-19 will aid in better understanding of the scenario. | This has been provided in the study area to provide context to the reader. ***[Lines 72-84]*** |
| Review 2 |  |  |
|  | PAGE 8 - LINE 11 - we can remove the word "got sick' in bracket | The correction has been effected as suggested. ***[Line 44]*** |
|  | In discussion, kindly add few points on the necessity of having Advanced labs in developing countries to get the genome sequencing done so that we can easily assess who will respond to which Cocktail antibody therapy | Thank you for this suggestion. The authors have added a few more comments to buttress the need for genomic sequencing. ***[Lines 249-253]*** |
