# Supplementary material for: COVID-19 reinfection in Liberia: Implication for improving disease surveillance
Source: PLoS One. 2022 Mar 24;17(3):e0265768. doi: 10.1371/journal.pone.0265768 (PMC8947140; doi:10.1371/journal.pone.0265768)
Supplement: Supplementary file 2 [file pone.0265768.s002.pdf]

The Incidence Management System (IMS)

COVID-19 RESPONSE

Republic of Liberia
P.O. BOX 10-9009
1000 MONROVIA 10, LIBERIA

OFFICE OF THE
INCIDENT MANAGER – COVID -19 RESPONSE
Ref. No.: 058

Dr. Wilhemina Jallah
Incident Manager, COVID -19 Response
Minister of Health
Republic of Liberia

August 1, 2020

Dr. Maame Amo- Addae
Resident Advisor
Liberia Field Epidemiology Training Program

Approval To Conduct the COVID-19 Response Related Research

Dear Dr. Amo- Addae,

I have the pleasure to inform you that following review and scrutiny of your COVID-19 response research activities as presented in the IMS meeting, the COVID-19 Incident Management System leadership for COVID-19 Pandemic Response in Liberia has endorsed and granted you approval for the underlisted study protocols. You are therefore advised to proceed to the field. The findings from the studies are expected to inform the current pandemic response in the country. The approval is to last for a period of one year from the date of this late. Do ensure that the informed consent form has the approval reference.
Upon completion of the study ensure that the findings and copy of the write up is presented to the IMS.
Be informed that no changes are permitted in the protocol without prior approval of the committee. The committee reverses the right to conduct compliance visits to your research site without prior notifications.
The studies that were authorized were:

1. COVID-19 Vaccine Hesitancy among adults in Liberia, April – May, 2021
2. Evaluation of Preparedness and Response to COVID-19 Pandemic in Liberia, August 2020
3. Routine health care services disruption during the first wave of COVID-19 epidemic, Liberia, August 2020
4. COVID-19 Reinfection in Liberia: Implication for improving disease surveillance, March 2020- July 2021

Sincerely yours,

Dr. Wilhemina Jallah

MINISTRY OF HEALTH
MINISTER
REPUBLIC OF LIBERIA
